# The Correlation between Serum Uric Acid and Renal Function in Elderly Chinese Diabetes with Normoalbuminuria

**DOI:** 10.1155/2019/1435875

**Published:** 2019-04-03

**Authors:** Qiaojing Qin, Yingjun Qian, Guanghua Zhu, Weifeng Fan, Jianying Niu, Yong Gu

**Affiliations:** ^1^Department of Nephrology, The Fifth People's Hospital of Shanghai, Fudan University, 200240, China; ^2^Jiangchuan Community Health Service Centre, Minhang District, Shanghai 200240, China; ^3^Department of Nephrology, Huashan Hospital, Fudan University, Shanghai 200040, China

## Abstract

**Objective:**

The elder diabetic patients increases rapidly in China and often accompany with hyperuricemia. Recently evidences show that renal function has been impaired in part of diabetic patients with normoalbuminuria. Therefore, we investigated the relationship between serum uric acid (SUA) and renal function in Chinese elder diabetes with normoalbuminuria.

**Methods:**

The physical examination data from 1052 cases of diabetic residents with normoalbuminuria aged 70 years and over in the Jiangchuan community of Minhang District, Shanghai, from October 2011 to September 2014 was analyzed retrospectively. Each received height, body weight, waist circumference (WC), waist-to-hip ratio (WHR), blood pressure (BP), and collected samples of fasting blood and morning urine to detect blood routine, blood glucose, glycosylated hemoglobin (HbA1c), blood lipids, serum creatinine, urinary albumin, urine creatinine, and urine PH value. Correlation between SUA and renal function, an index of which is estimated using estimated glomerular filtration rate (eGFR), was analyzed.

**Results:**

The prevalence of hyperuricemia was 21.10%. Levels of WC and triglyceride (TG) increased and the levels of HbA1c, high density lipoprotein-cholesterol (HDL-C), eGFR, and urine PH decreased while the levels of SUA increased. Moreover, negative correlation of eGFR with age, WC, leukocyte, and SUA (Pearson r=0.415) was observed via Pearson correlation analysis. It indicates the strong association between SUA and eGFR. Furthermore, eGFR independently associated with SUA, age, leukocyte, hemoglobin (Hb), and fasting blood glucose (FBG) was confirmed by multiple linear stepwise regression analysis.

**Conclusion:**

SUA may play an important role in the decrease of eGFR in elderly Chinese diabetic patients with normoalbuminuria.

## 1. Introduction

Diabetes mellitus (DM) is a metabolic disease characterized by an increase in chronic blood glucose levels due to defects in insulin secretion and action [1], causing multisystem damage [2–4]. Epidemiological studies have shown that the current prevalence of diabetes in China is 9.7%, and the number of diabetic patients is over 92 million [5]. In Shanghai, China, life expectancy is obvious prolonged accompany with rapid economic development [6]. Although the management on blood glucose, blood lipids, and blood pressure in diabetic patients has been strengthened, diabetes has become the primary cause of new dialysis patients in Shanghai, China [7].

Recently, studies provides evidence to support that kidney function has been impaired in part of diabetes in which urinary albumin creatinine ratio (UACR) are normal [8] and the diabetic patients are often accompanied with hyperuricemia [9], knowledge regarding the relationship between serum uric acid (SUA), and kidney function in Chinese diabetes with normoalbuminuria over 70 years old is still unclear.

Therefore, using the complete physical examination data from 1052 case from October 2011 to September 2014 in Chinese diabetic patients with normoalbuminuria over 70 years old in Jiangchuan Community, Minhang District, Shanghai, China, we retrospectively analyze the relationship between SUA and estimated glomerular filtration rate (eGFR) to explore the effect of SUA on the decline of renal function in elderly diabetic patients with normoalbuminuria. It may be to provide the theoretical basis for effective public health policy measures for elderly diabetes.

## 2. Materials and Methods

### 2.1. Subjects

The Jiangchuan Community is located in the southern part of Minhang District, Shanghai, China. Free medical physical examination service for elderly patients who had been diagnosed with diabetes and lived in the Jiangchuan Community. The diagnosis of diabetes had been based on plasma glucose (PG) criteria, either fasting PG≥ 7.0mmol/L, and/or 2-h PG≥11.1, or self-reported history of diabetes.

From October 2011 to September 2014, total of 1052 diabetic patients, 478 males and 574 females, with ACR in the normal range who accepted questionnaire and free medical measurement were included in the study. These patients were aged from 70 to 92 with an average age of 75.59 ± 4.32 years old. The study was approved by the Ethics Committee of the Fifth People's Hospital of Shanghai, Fudan University.

### 2.2. Data Collection

The survey included three parts: questionnaire, physical measurement, and laboratory examination. First, the questionnaire was complete. Subsequently, blood and urine specimen were collected after physical measurement had been finished in all subjects fasted for 12 hours in the morning. Data were collected by the trained investigator: (1) Questionnaire survey: including the demographic characteristics of the subjects (name, gender, birth date, place of residence, ethnicity, and personal disease history (including hypertension, diabetes)). (2) Physical measurement: height, weight, waist circumference, hip circumference, and blood pressure were measured according to the recommendations by the World Health Organization. (3) Laboratory examination: levels of hemoglobin and leukocyte were measured by globulimeter, blood glucose by glucose oxidase method, glycosylated hemoglobin (HbA1c) by high performance liquid chromatography, blood lipids and serum creatinine by autoanalyzer, and urinary albumin by immunoturbidimetry. At the same time, level of urinary creatinine was assayed using the same method as that for serum creatinine. (4) Evaluation of GFR (eGFR): the eGFR value was calculated according to the CKD-EPI formula: eGFR [ml·min^−1^·(1.73m^2^)^−1^] = 141 × (Scr/K)^a^ × (0.993)^age^ × 1.018 (female). The K value in the formula: female = 62, male = 80. The a value in the formula is as follows: female Scr ≤ 62 then a = -0.329, Scr > 62 then a = -1.209; male Scr ≤ 80 then a = -0.41l, and Scr > 80 then a = - 1.209. The unit of serum creatinine is mg/dl, and the age is years. (5) Relevant definitions: hyperuricemia [10]: serum uric acid ≥ 420 umol /L in male, ≥ 360 umol / L in female. Normoalbuminuria: according to UACR, it was normal albuminuria (ACR<30 mg/g) [11].

### 2.3. Statistical Analysis

Statistical analysis was performed using SPSS 22.0 software for Windows. Measurement data conforming to normal distribution and homogeneity of variance were presented as mean  ±  standard deviation (SD), and the one-way ANOVA and T test were used. Linear correlations between parameters were tested with Pearson correlation and multiple linear stepwise regression analysis. The null hypothesis was rejected when p < 0.05.

## 3. Results

### 3.1. General Clinical Data

A total of 1052 community residents aged 70-92 entered the study, including 478 males, accounting for 45.44%, age (75.56 ± 4.44) years old; 574 females, accounting for 54.56%, age (75.61 ± 4.23) years old. The prevalence rate of hyperuricemia was 16.53% for men and 24.91% for women, and the overall prevalence rate was 21.10% (Table 1).

Subsequently, the subjects were grouped according to serum uric acid quartile. As the serum uric acid (SUA) increased, the levels of waist circumference (WC), waist-to-hip ratio (WHC), body mass index (BMI), and total triglyceride (TG) were increased gradually in male while the levels of fasting blood glucose (FBG), glycated hemoglobin (HbA1c), lipoprotein-cholesterol (HDL-C), eGFR, and urine PH were gradually decreased (Table 2). In female, with the increase of SUA, age, diastolic blood pressure (DBP), leukocyte, WC, TG, and total cholesterol (TC) levels increased significantly, while the levels of FBG, HbA1c, HDL-C, eGFR, and urine PH value gradually decreases (Table 3).

Especially, with the increase of SUA, eGFR levels were decreased significantly in males and females. eGFR levels of male patients in group Q2, Q3, and Q4 were significantly lower than those in Q1 group, while eGFR levels in Q4 group were lower than those in Q2 group (Table 2). In female patients, eGFR levels in group Q3 and Q4 were significantly lower than those in Q1 and Q2 group, while eGFR levels in Q4 group were lower than those in Q3 group (Table 3). The results suggest that SUA may be an important factor effecting on eGFR. There were no significant differences in the levels of UACR in female or male groups (Tables 2 and 3).

### 3.2. Correlation Analysis between eGFR and SUA

To investigate the correlation between eGFR and parameters, we used Pearson correlation analysis and found negative correlation between eGFR and SUA (Pearson r = -0.415, P<0.001). In males, eGFR was negatively correlated with age, leukocyte, and SUA, positively correlated with FBG, HbA1c, HDL-C, and urine PH value, and strongly correlated with age (Pearson r = -0.355, P<0.001) and SUA (Pearson r = -0.396, P<0.001). Similarly, in female, eGFR was negatively correlated with age, leukocyte, SUA, and urine PH value, positively correlated with Hb, and strongly correlated with age (Pearson r= -0.454, P<0.001) and SUA (Pearson r= -0.422, P<0.001) (Table 4).

Furthermore, we investigated the possible relation between eGFR and SUA using multiple linear stepwise regression analysis. It showed that eGFR was independently associated with SUA whether male, female, or total patients, and the standardized regression coefficient was -0.348, -0.347, and -0.367, respectively. In addition, age, leukocyte, Hb, and FBG were independently correlated with eGFR in total patients, and the standardized regression coefficients were -0.363, -0.086, 0.069, and 0.055, respectively. But Hb in male and FBG in female patients did not show the association with eGFR although age and leukocyte are associated with eGFR in male or female patients (Tables 5, 6, and 7).

## 4. Discussion

With the increase in life expectancy and the large number of diabetic patients, diabetes has replaced chronic glomerulonephritis as the primary cause of new dialysis patients in Shanghai [7] and Beijing [12] which are the big cities in China although the control of blood glucose, blood lipids, and blood pressure [13–15] has been strengthened. Recently, studies have shown that a significant proportion of diabetes with normoalbuminuria have glomerular filtration rate (GFR) decrease [8, 16] while serum uric acid (SUA) has reentered the spotlight as a cause of renal insufficiency [17, 18].

Most diabetic patients reside in the community. The physical examination data from community diabetes patients may better represent the real situation of these patients. Therefore, we investigated the association SUA with renal function via complete physical examination data from diabetic patients aged 70 years and over without albuminuria in Jiangchuan community, Shanghai, from October 2011 to September 2014.

There were total of 1052,478 male and 574 female, elder diabetic patients in our study. The results show that the prevalence of hyperuricemia is 16.53% in male, 24.91% in female, and 21.10% in total patients. It showed a high prevalence of hyperuricemia which is significantly higher in elderly diabetic patients with normoalbuminuria than in the developing or developed country [19–21].

The prevalence of hyperuricemia in female is 24.91%, which is higher than 16.53% in male, which is inconsistent with previous studies [22]. Estrogen has the excretion effect of uric acid in women, so the prevalence of hyperuricemia in men is higher than that in female [23]. In our survey, the patients were all over 70 years old, and the decreased estrogen in female patients may be the reason for the higher prevalence of hyperuricemia in female than in male.

Currently, serum creatinine (SCr) and GFR are commonly used indicators in clinical renal function [24]. EPI formula is used to calculate estimated glomerular filtration rate (eGFR) values [24, 25] to evaluate renal function in our study. The patients were divided into four groups according to gender and the quartile of SUA. Accompanying with the SUA increase, levels of eGFR were gradually decreased in all male and female patients. At the same time, the waist circumference, waist-to-hip ratio, BMI, and TG levels gradually increased while FBG, HbA1c, HDL-C, and urine PH values gradually decreased in male patients. Age, diastolic blood pressure, waist circumference, leukocyte, TC, and TG levels gradually increased while HbA1c, HDL-C, and urine PH value gradually decreased in female patients. These results indicate that SUA may be correlated with eGFR, waist circumference, waist-to-hip ratio, BMI, TC, TG, FBG, HbA1c, HDL-C, and urine PH value.

Subsequently, the correlation between eGFR and various parameters was analyzed via Pearson linear correlation analysis. The results showed that eGFR was negatively correlated with age, leukocyte, and SUA and positively correlated with FBG, HbA1c, HDL-C, and urine PH value in male. Moreover, eGFR is negatively correlated with age, leukocyte, and SUA and positively correlated with hemoglobin in female. eGFR especially highly correlated with age (r=-0.355 in male, r=-0.454 in female) and SUA (r=-0.396 in male, r=-0.422 in female). These results show that eGFR strongly correlates with SUA in both male and female patients.

Hyperuricemia has been shown to be an independent risk factor for renal injury [26]. In our study, the relation between eGFR and SUA was analyzed via multiple linear stepwise regression analysis. The result showed that eGFR was independently associated with SUA in male, female, or total patients. It also confirmed that SUA play an important role in the decline of eGFR in diabetic patients with normoalbuminuria over 70 years old. The mechanism may be as follows. (1) Uric acid induces endothelial dysfunction via the impairment of nitric oxide synthesis [27]. (2) Uric acid in the dissolved state can stimulate the inflammatory response via inducing the production of chemokine such as monocyte chemoattractant protein [28]. Our results also showed the negative correlation between leukocyte and eGFR.

There was no association significantly between UACR or blood pressure and eGFR. It may be the results from the objects with normal UACR including nonhypertensive patients and do not deny the effects of blood pressure and ACR on eGFR of diabetes.

FBG and HbA1c were found to be positively correlated with eGFR in the linear correlation analysis. Multivariate stepwise regression analysis showed that only eGFR was independently associated with FBG. This result may be due to the fact that the study population we included was diabetic patient with normoalbuminuria in which hyperglycemia promoted the increase of eGFR [29] and need more patients to elaborate the relation between HbA1c and eGFR.

Han [30] reported that eGFR is associated with Hb Level in Korean Adults. Our study also showed Hb in female associated with eGFR independently. The main limitation is not enough patients in this study to result in certain bias of results that may exist. In future, we will further study the effects of various factors such as Hb, FBG, antihypertensive drugs, and hypoglycemic agents on GFR in large diabetic patients.

## 5. Conclusions

This study for the first time shows that hyperuricemia accounted for 21.1%, and SUA play an important role in the decline of eGFR in elderly Chinese diabetic patients with normoalbuminuria. It suggests that hyperuricemia should be controlled effectively to inhibit the GFR decrease and protect the renal function in these patients. Due to the large number of patients, Chinese government's public health policy should strengthen the control of hyperuricemia as soon as possible in community elder nationality diabetes with normoalbuminuria.

## Figures and Tables

**Table 1:** General clinical characteristics of hyperuricemia in patients.

Parameter	N (%)	Age (years)
Total	1052	75.59 ± 4.32
hyperuricemia	222 (21.10%)	76.35 ± 4.27
male	478 (45.44%)	75.56 ± 4.44
hyperuricemia	79 (16.53%)	75.87 ± 4.51
Female	574 (54.56%)	75.61 ± 4.23
hyperuricemia	143 (24.91%)	76.65 ± 4.10

**Table 2:** Clinical data in male patients according to SUA quartile.

Parameter	Q1 group	Q2 group	Q3 group	Q4 group	P value
	(SUA≤295)	(347≥SUA≥296)	(406≥SUA≥348)	(SUA≥407)	
n	124	116	119	119	
Age (years)	75.08 ± 4.67	75.5 ± 4.53	75.44 ± 3.90	75.92 ± 4.61	0.917
SBP (mmHg)	134.13 ± 15.94	133.43 ± 13.56	137.22 ± 20.82	135.22 ± 14.36	0.309
DBP (mmHg)	79.52 ± 8.84	79.81 ± 8.37	80.21 ± 8.11	80.16 ± 7.44	0.378
WC (cm)	85.25 ± 8.03	86.06 ± 8.46	87.73 ± 8.27*∗*	87.97 ± 9.41*∗*	0.037
WHR	0.89 ± 0.05	0.91 ± 0.05	0.92 ± 0.06*∗*	0.92 ± 0.07*∗*	0.003
BMI (kg/m^2^)	24.24 ± 3.07	24.34 ± 2.81	25.08 ± 3.12	25.35 ± 3.10*∗*	0.009
FBG (mmol/L)	8.79 ± 3.10	7.79 ± 2.78*∗*	7.50 ± 1.73*∗*	7.43 ± 1.59*∗*	<0.001
HbA1c(%)	7.69 ± 2.24	7.36 ± 1.89*∗*	6.99 ± 1.33*∗*	6.92 ± 1.17*∗*	<0.001
TC (mmol/L)	4.67 ± 0.86	4.66 ± 0.95	4.58 ± 1.05	4.79 ± 0.89	0.382
TG (mmol/L)	1.27 ± 0.62	1.53 ± 1.07	1.50 ± 0.65*∗*	1.97 ± 1.08*∗*	<0.001
LDL-C (mmol/L)	2.78 ± 0.63	2.76 ± 0.65	2.68 ± 0.82	2.83 ± 0.65	0.426
HDL-C (mmol/L)	1.27 ± 0.27	1.21 ± 0.25	1.19 ± 0.29	1.13 ± 0.19*∗*	<0.001
eGFR (ml/min/1.73m^2^)	89.95 ± 8.01	88.80 ± 9.32*∗*	82.51 ± 10.47*∗*	78.70 ± 12.72*∗*^∆^	<0.001
leukocyte (x109/L)	6.17 ± 1.57	6.04 ± 1.40	6.47 ± 1.55	6.39 ± 1.57	0.105
Hb(g/L)	143.31 ± 13.12	145.11 ± 15.22	144.40 ± 12.61	145.24 ± 13.24	0.674
SUA(*μ*mol/L)	258.71 ± 27.45	322.25 ± 15.66*∗*	376.76 ± 17.83*∗*^∆^	456.53 ± 57.28*∗*^∆#^	<0.001
Urine pH	5.98 ± 0.85	5.73 ± 0.67	5.68 ± 0.76*∗*	5.61 ± 0.69*∗*	0.001
UACR	10.73 ± 7.03	9.06 ± 6.69	9.08 ± 6.71	10.37 ± 8.13	0.154

Compared with Q1 group, *∗*P<0.05; compared with Q2 group, ^∆^P<0.05; compared with Q3 group, ^#^P<0.05; systolic blood pressure (SBP), diastolic blood pressure (DBP), waist circumference (WC), waist-to-hip ratio (WHR), body mass index (BMI), fasting blood glucose (FBG), glycated hemoglobin (HbA1c), triglyceride (TG), total cholesterol (TC), low density lipoprotein-cholesterol (LDL-C), high density lipoprotein-cholesterol (HDL-C), hemoglobin (Hb), serum uric acid (SUA), and urinary albumin creatinine ratio (UACR).

**Table 3:** Clinical data in female patients according to SUA quartile.

Parameter	Q1 group	Q2 group	Q3 group	Q4 group	P value
	(SUA≤266)	(309≥SUA≥267)	359≥SUA≥310	(≥360)	
n	150	139	142	143	
Age (years)	75.35 ± 4.40	75.03 ± 3.90	75.41 ± 4.34	76.65 ± 4.11*∗*^∆^	0.007
SBP (mmHg)	133.69 ± 14.39	135.99 ± 15.38	136.37 ± 16.74	138.50 ± 14.91	0.066
DBP (mmHg)	78.31 ± 7.46	78.50 ± 7.90	80.01 ± 7.82	80.78 ± 7.39*∗*	0.015
WC (cm)	82.11 ± 8.55	82.44 ± 8.52	83.09 ± 8.37	85.36 ± 8.46*∗*^∆^	0.005
WHR	0.88 ± 0.06	0.88 ± 0.06	0.88 ± 0.07	0.89 ± 0.06	0.557
BMI (kg/m^2^)	24.15 ± 3.47	24.73 ± 3.34	24.78 ± 3.20	25.83 ± 3.31*∗*^∆^	<0.001
FBG (mmol/L)	8.39 ± 2.84	7.48 ± 1.91*∗*	7.62 ± 2.08	7.65 ± 1.94	0.002
HbA1c(%)	7.56 ± 1.99	6.93 ± 1.39*∗*	6.88 ± 1.29*∗*	7.00 ± 1.35*∗*	<0.001
TC (mmol/L)	5.23 ± 1.00	5.04 ± 1.03	5.20 ± 0.92	5.41 ± 1.06^∆^	0.021
TG (mmol/L)	1.56 ± 0.70	1.70 ± 1.39	1.85 ± 1.07*∗*	2.17 ± 1.49*∗*^∆^	<0.001
LDL-C (mmol/L)	2.99 ± 0.67	2.89 ± 0.74	3.02 ± 0.61	3.09 ± 0.79	0.132
HDL-C (mmol/L)	1.37 ± 0.30	1.34 ± 0.28	1.26 ± 0.25*∗*	1.25 ± 0.25*∗*^∆^	<0.001
eGFR (ml/min/1.73m^2^)	90.01 ± 8.10	88.75 ± 6.49	85.63 ± 11.41*∗*^∆^	78.75 ± 14.42*∗*^∆#^	<0.001
Leukocyte (x10^9^/L)	6.03 ± 1.49	5.94 ± 1.36	6.34 ± 1.48	7.02 ± 1.86*∗*^∆#^	<0.001
Hb(g/L)	135.13 ± 11.41	134.41 ± 14.78	132.49 ± 10.23	133.08 ± 16.72	0.325
SUA(*μ*mol/L)	230.68 ± 25.69	288.58 ± 12.80*∗*	331.44 ± 14.67*∗*^∆^	416.80 ± 55.95*∗*^∆#^	<0.001
Urine pH	5.81 ± 0.75	5.67 ± 0.70	5.67 ± 0.72	5.58 ± 0.72*∗*	0.057
UACR	11.77 ± 7.27	11.23 ± 7.34	11.10 ± 7.57	12.31 ± 7.72	0.508

Compared with Q1 group, *∗*P<0.05; compared with Q2 group, ^∆^P<0.05; compared with Q3 group, ^#^P<0.05;

**Table 4:** Linear correlation between eGFR and parameters including SUA.

Variables	Total		Male		Female	
	(n=1052)		(n=478)		(n=574)	
	r value	P value	r value	P value	r value	P value
Age	-0.407	<0.001	-0.355	<0.001	-0.454	<0.001
SBP	0.039	0.207	-0.028	0.545	-0.054	0.196
DBP	0.003	0.919	-0.019	0.685	-0.013	0.765
WC	-0.066	0.031	-0.042	0.360	-0.060	0.151
WHR	-0.064	0.038	-0.042	0.361	-0.055	0.189
BMI	-0.045	0.142	-0.047	0.307	-0.047	0.262
FBG	0.115	<0.001	0.180	<0.001	0.065	0.122
HbA1c	0.105	<0.001	0.146	0.001	0.073	0.082
TC	0.031	0.319	-0.012	0.790	0.027	0.518
TG	-0.027	0.381	-0.085	0.062	-0.008	0.857
LDL-C	0.004	0.887	-0.028	0.544	0.006	0.881
HDL-C	0.092	0.003	0.097	0.034	0.064	0.125
Leukocyte	-0.154	<0.001	-0.112	0.014	-0.189	<0.001
Hb	0.081	0.008	0.040	0.382	0.182	<0.001
SUA	-0.415	<0.001	-0.396	<0.001	-0.422	<0.001
Urine PH	0.098	0.002	0.130	0.004	-0.079	0.060
UACR	-0.034	0.272	-0.005	0.905	-0.073	0.079

**Table 5:** Multiple linear stepwise regression analysis for association between eGFR and SUA in all patients.

variables	B	SE	Beta	t	P value
(constant)	167.924	6.117		27.453	P<0.001
SUA	-0.052	0.004	-0.367	-13.977	P<0.001
Age	-0.946	0.067	-0.363	-14.103	P<0.001
Leukocyte	-0.612	0.186	-0.086	-3.296	0.001
Hb	0.054	0.020	0.069	2.680	0.007
FBG	0.270	0.127	0.055	2.119	0.034

Serum uric acid (SUA), hemoglobin (Hb), and fasting blood glucose (FBG).

**Table 6:** Multiple linear stepwise regression analysis for association between eGFR and SUA in male patients.

variables	B	SE	Beta	t	P value
(constant)	163.498	7.917		20.650	P<0.001
SUA	-0.048	0.006	-0.348	-8.652	P<0.001
Age	-0.823	0.097	-0.331	-8.477	P<0.001
FBG	0.432	0.190	0.091	2.273	0.023
Leukocyte	-0.623	0.283	-0.086	-2.206	0.028

**Table 7:** Multiple linear stepwise regression analysis for association between eGFR and SUA in female patients.

variables	B	SE	Beta	t	P value
(constant)	171.976	8.218		20.926	P<0.001
Age	-1.063	0.091	-0.394	-11.642	P<0.001
SUA	-0.052	0.005	-0.347	-9.947	P<0.001
Hb	0.107	0.029	0.127	3.753	P<0.001
Leukocyte	-0.574	0.247	-0.081	-2.323	0.021

## Data Availability

The data analysed during the study are not publicly available, in order to protect patient privacy, as it might be possible to identify the results of an individual patient from this limited group of patients.
